# Associations of Happiness, *Ikigai*, and Enjoyment of Daily Life with Frequency of Forest Walking in a Japanese General Population: The Japan Multi-Institutional Collaborative Cohort Daiko Study

**DOI:** 10.3390/ijerph23081020

**Published:** 2026-08-04

**Authors:** Emi Morita, Sayo Kawai, Tae Sasakabe, Rieko Okada, Yoko Kubo, Yoko Mitsuda, Yasufumi Kato, Takashi Matsunaga, Mako Nagayoshi, Takashi Tamura, Kenji Wakai

**Affiliations:** 1 Forestry and Forest Products Research Institute, Forest Research and Management Organization, 1 Matsunosato, Tsukuba 305-8687, Ibaraki, Japan; 2 International Institute for Integrative Sleep Medicine (WPI-IIIS), University of Tsukuba, 1-1-1 Tennodai, Tsukuba 305-8577, Ibaraki, Japan; 3 Department of Public Health, Aichi Medical University School of Medicine, Nagakute 480-1195, Aichi, Japan; 4 Department of Preventive Medicine, Nagoya University Graduate School of Medicine, 65 Tsurumai-Cho, Showa-ku, Nagoya 466-8550, Aichi, Japanmatsunaga.takashi.b8@f.mail.nagoya-u.ac.jp (T.M.); nagayoshi.mako.z3@f.mail.nagoya-u.ac.jp (M.N.); ttamura@med.nagoya-u.ac.jp (T.T.); wakai.kenji.y2@f.mail.nagoya-u.ac.jp (K.W.)

**Keywords:** *shinrin-yoku*, forest bathing, *ikigai*, well-being, happiness, public health, epidemiology, general population, J-MICC Study

## Abstract

A number of studies have examined the acute effects of *shinrin-yoku* (forest bathing), or forest walking; however, only a limited number of epidemiological studies have examined the association between habitual forest walking and daily health. The aim of this study was to examine the association of daily happiness, the Japanese concept of *ikigai*, and enjoyment of daily life with the frequency of forest walking. We used data from the secondary survey of the Japan Multi-Institutional Collaborative Cohort (J-MICC) Daiko study for this cross-sectional study. A total of 3472 participants (2518 women) were included in the analysis. Logistic regression analysis showed that the aORs for frequency of feeling happy, degree of happiness, *ikigai*, and daily life enjoyment were 1.72 (95% confidence interval [CI]: 1.29–2.28), 1.54 (95% CI: 1.14–2.09), 1.65 (95% CI: 1.25–2.20), and 2.38 (95% CI: 1.60–3.35), respectively, for those who reported going for forest walks at least once per month when those who reported rarely engaging in this practice served as the reference. This study found that the frequency of forest walking was associated with daily happiness, *ikigai*, and enjoyment of daily life, suggesting that occasional forest bathing may lead to improved well-being and quality of life.

## 1. Introduction

Exposure to forest environments has been reported to exert positive psychological and physical effects on the human body. Japan has one of the highest proportions of forested areas in the world, with forests accounting for 67% of its land compared with the global average of 31% [1]. In Japan, with abundant forest resources, forest walking or “*shinrin-yoku*” (walking and/or staying in forested areas) is a well-known activity for health promotion using forest environments. *Shinrin* means forests in Japanese, while yoku means to be exposed to either hot water or fresh air; hence, *shinrin-yoku* can also be translated as “forest bathing.” *Shinrin-yoku* was first proposed in Japan in the 1980s and is considered to promote both physical and mental health through the inhalation of substances released from trees, exercise, and other healing factors associated with the forest environment [2]. Forest bathing is a method of promoting health wherein the intensity of the exercise does not matter and no special skills are required, as the focus is on exposure to the forest environment. Therefore, individuals who walk or stay in a forest environment are considered to be practicing forest bathing.

According to a public opinion poll conducted by the Cabinet Office in Japan in 2023, 44.9% of individuals who had visited a forest in the past year said that their purpose for visiting the forest was to refresh themselves through the healing effects of the forest via forest bathing [3], indicating that the practice is widespread in the country.

Although the concept of *shinrin-yoku* originated in Japan, it has grown and has also become a widespread practice in many other countries over recent years. A number of studies have been conducted on the effects of forest bathing, forest walking, and comprehensive forest therapy programs involving overnight stays. Most such studies have used interventions to examine the acute effects of engaging in just a single session of forest walking, forest bathing, or forest therapy with a relatively small number of participants [4,5,6,7]. One meta-analysis of blood pressure reported that forest environments significantly lowered blood pressure compared with their non-forest counterparts [6]. Psychological benefits (e.g., reduced anxiety) [4,7], lower levels of stress-related hormones (e.g., cortisol) [8], improved sleep on nights following forest walks [9], and beneficial changes regarding immunological and inflammatory parameters [4] have also been reported. A study of over 500 people, examining factors related to acute psychological effects, reported that the effect of forest walks in terms of reducing anxiety was greater in people who experienced chronic stress, but that there was no association with the distance walked or length of stay [10]. Another large online survey also reported that well-being increased after guided forest bathing with nature connection activities [11].

These findings show that a single forest bathing session can have temporary effects. From the perspective of public health, however, one-session healthy actions (e.g., one session of healthy eating or exercising) do not generally lead to daily health promotion or disease prevention because the temporary effects dissipate over time. Health promotion and disease prevention can be achieved by continually practicing healthy behaviors.

To evaluate the association between regular forest bathing and health, population-based epidemiological studies are necessary. However, although a number of epidemiological studies have examined the association between green spaces and health [12,13,14,15], only a few studies have investigated the association between regular forest bathing or forest walks and health. According to these epidemiological studies, the more often people go for forest walks, the lower the proportion of insomnia symptoms among women, and the higher the proportion of high stress coping ability [16,17]. On the other hand, although blood pressure has been reported to decrease slightly after a single session of forest bathing in a meta-analysis [6], two epidemiological studies showed that the frequency of forest walking was not associated with the prevalence of hypertension [18,19]. The studies suggested that forest walking, which does not require a certain level of exercise intensity, just once a week, may be ineffective for preventing or improving hypertension. This suggests that results regarding acute, temporary effects are not necessarily consistent with those of epidemiological studies. Therefore, even if acute effects are observed, the effects of regular forest bathing on daily health should be verified through epidemiological studies.

Furthermore, while studies have examined the association between a connection with nature and feelings of happiness [20], few have investigated the association between regular forest bathing and daily well-being such as happiness and enjoyment of daily life. Furthermore, the association between forest bathing and “*ikigai*” remains unclear.

*Ikigai* is a Japanese concept that is used in everyday life, and it has become known in other countries in recent years [21]. A Japanese dictionary defines “*ikigai*” as “something worth living for; the motivation and joy of living [22].” In papers, it is sometimes translated as “purpose in life”, “reason for living”, “a sense of life worth living”, or “that which most makes one’s life seem worth living” [21,23,24,25,26]. Among Japanese people, happiness is more closely associated with hedonic well-being, whereas *ikigai* is considered conceptually similar to eudaimonic well-being [27].

One paper described *Ikigai* as the small joys and a deep sense of meaning that make life worthwhile, as well as feelings rooted in everyday experience, community, and personal values rather than career success [28]. However, what gives people *ikigai* from person to person includes both of these aspects. While many people find *ikigai* in the small joys of daily life, such as family or hobbies [29], others find their *ikigai* through their work and devote themselves to it [30].

A higher sense of *ikigai* has been associated with fewer depressive and anxiety symptoms, higher health-related quality of life (QOL), higher happiness levels, and higher satisfaction with life levels [31]. It has also been reported that individuals with a sense of *ikigai* have a lower risk of all-cause mortality, mortality from cardiovascular diseases in men, cardiovascular diseases, and both developing dementia and developing functional disability among older adults [32,33,34]. Thus, *ikigai* is related to individuals’ health, happiness, and QOL and is important to ensure a good life. However, to the best of our knowledge, no studies have thus far examined the association between the practice of forest bathing and *ikigai* through epidemiological studies.

Based on the foregoing, this study aimed to examine the associations among individuals’ frequency of forest walking and their daily happiness, *ikigai*, and enjoyment of daily life in a large Japanese general population.

## 2. Methods

### 2.1. Study Procedures

The study design of this article was a cross-sectional study. This study used data from the second survey of the Daiko Study, a component of the Japan Multi-Institutional Collaborative Cohort Study. The J-MICC Study is a long-term genomic cohort study that aims to verify the interactions among genetic factors, lifestyle, and lifestyle-related diseases, particularly cancer. The baseline survey of the Daiko Study was conducted between 2008 and 2010. Participants were Nagoya residents aged 35–69 years at the time of the survey. The protocol for the J-MICC Daiko Study and frequency of forest walking found in the baseline survey have both been reported previously [35,36].

The second survey, from which the data used in this study was obtained, was conducted in 2014, approximately five years after the baseline survey, among participants of the baseline survey who agreed to participate again. This study used only data from the second survey of the Daiko Study because the second survey included questions regarding *ikigai* and happiness.

The city of Nagoya is located in the center of the main island of Japan. It is a large city with a population of over 2 million, and the percentage of forested areas in the city is only 2.9% [37]. The percentage of forested areas in Aichi Prefecture, in which Nagoya City is located, is 42% [38].

### 2.2. Participants

Those who did not respond to the survey items on their frequency of forest walking, happiness, *ikigai*, lifestyle, enjoyment of life, and other items relevant to this study were excluded. In total, 3472 participants (954 men, 2518 women), with an average age of 58.7 ± 10.0 years, were included in the analysis.

### 2.3. Questionnaire

The participants were requested to complete a self-administrated questionnaire assessing the frequency of forest walking as an exposure to the forest environment. The question was “How frequently do you go forest walking, including hiking, nature observation, mountain-walking, and working or camping in forested areas, but excluding visits to city parks?” The six possible responses were as follows: 1 (once a week or more), 2 (two or three times a month), 3 (once a month), 4 (several times a year), 5 (once a year), and 6 (rarely). These six categories were modified into four categories in the analysis by combining options (1), (2), and (3) into one category termed “once a month or more.” Thus, the categories were once a month or more, several times a year, once a year, and rarely.

The participants were also asked about their working status (working, including employees, employers, and self-employed individuals/not working), years of education, and lifestyle factors such as smoking status, alcohol consumption, and leisure-time activities (including the intensity, frequency, and duration of exercise).

The outcome measures were assessed using four questions on the participants’ frequency and degree of happiness, *ikigai*, and self-rated enjoyment of daily life. Although the outcome measures were assessed on a four-point or five-point scale, they were dichotomized for logistic regression analysis in the statistical analysis. In this study, the top two response options were defined as “Yes.” First, the frequency of happiness was measured through the question: “Over the past few weeks, have you generally felt happier than usual?” The response options were 1 (often), 2 (yes), 3 (no), and 4 (never). Those who answered 1 or 2 were defined as having experienced feeling happier than usual (“experience of feeling happier” hereafter). The degree of happiness was measured through the question: “Overall, how happy do you feel?” The response options were 1 (very happy), 2 (happy), 3 (undecided), 4 (unhappy), and 5 (very unhappy). Those who answered 1 or 2 were defined as having a sense of happiness. Second, *ikigai* was measured through the question: “Do you have *ikigai*?” The response options were 1 (yes, very much), 2 (yes), 3 (not very much), and 4 (not at all). Those who answered 1 or 2 were defined as having *ikigai*. Third, self-rated enjoyment of daily life was assessed using the question: “Are you enjoying your daily life?” The response options were 1 (I always enjoy it), 2 (I enjoy it most of the time), 3 (I don’t enjoy it much), and 4 (I do not enjoy it at all). Participants who answered 1 or 2 were classified as having enjoyment of daily life.

Among the lifestyle factors, habitual alcohol consumption was defined as once a week or more. Habitual exercise was defined as ≥30 min of leisure time activities at least once a week; the intensity of the exercise was not considered [18].

### 2.4. Statistical Analysis

Differences in proportions were assessed using the chi-squared test. The Mantel–Haenszel test of trends was used to assess trends in the proportions of the ordinal variables. In logistic regression analysis, age and the lifestyle factors were adjusted for in Model 1. The dependent variables in the model were having experience of feeling happier (yes/no), a sense of happiness (yes/no), *ikigai* (yes/no), and enjoyment of daily life (yes/no), while the independent variables in Model 1 were age (≤49, 50s, 60s, and ≥70 [years]), frequency of forest walking (four categories), smoking status (current smoker/other), alcohol consumption (once a week or more/other), and habitual exercise (yes/no). For the entire study population, sex was also included as an independent variable. In Model 2, to consider social factors as well, we added whether the respondent had a working status and years of education as independent variables, in addition to those in Model 1.

Statistical significance was set at 5%. SPSS Statistics version 23 for Windows (IBM Corp., Armonk, NY, USA) was used to perform the statistical analyses.

## 3. Results

### 3.1. Participant Characteristics

For all participants, the numbers and proportions of the participants in each frequency category were as follows: once a month or more (*n* = 334, 9.6%), several times a year (*n* = 771, 22.2%), once a year (*n* = 391, 11.3%), and rarely (*n* = 1976, 56.9%). Table 1 presents the characteristics of the participants by their frequency of forest walking.

The frequency of forest walking differed significantly between men and women (chi-square test: *p* < 0.001), as the higher-frequency groups included a higher percentage of men. Among all participants, significant trends in the frequency of forest walking were observed by age (trend *p* < 0.001), alcohol consumption (trend *p* < 0.001), and habitual exercise (trend *p* < 0.001). Significant trends were also observed by alcohol consumption (*p* = 0.01) and habitual exercise (trend *p* < 0.001) among the men, as well as by age (trend *p* = 0.001), alcohol consumption (*p* = 0.02), and habitual exercise (*p* < 0.001) among the women. The inverse association between the frequency of forest walking and employed was observed among all participants (*p* < 0.001), both men (*p* < 0.001) and women (*p* = 0.03).

### 3.2. Associations of Happiness, Ikigai, and Enjoyment of Daily Life with Frequency of Forest Walking

The percentages of the participants who reported having experience of feeling happier than usual were 68.2% (*n* = 2368/3472) among all participants, 65.3% (*n* = 623/954) among the men, and 69.3% (*n* = 1745/2518) among the women. The percentages who reported having a sense of happiness were 75.9% (*n* = 2634/3472) among all participants, 74.6% (*n* = 712/954) among the men, and 76.3% (*n* = 1922/2518) among the women. The percentages who reported having *ikigai* were 68.8% (n = 2383/3472) among all participants, 72.1% (*n* = 688/954) among the men, and 67.3% (*n* = 1695/2518) among the women. The percentages who enjoy daily life were 81.2% (*n* = 2820/3472) among all participants, 80.2% (*n* = 765/954) among the men, and 81.6% (*n* = 2055/2518) among the women.

Table 2 presents the percentages of happiness, *ikigai*, and enjoyment of daily life by the frequency of forest walking. For all participants—as well as for both men and women—there was a statistically significant trend showing that the more frequently participants went for forest walks, the higher the percentage of experiencing feeling happier, sense of happiness, *ikigai*, and enjoyment of daily life; all trends *p* < 0.001, except for a sense of happiness in men (*p* = 0.03).

For men, although the results of the trend test were statistically significant, a clear relationship—whereby a higher frequency of forest walking is associated with a greater proportion of men reporting a sense of happiness, *ikigai*, and enjoyment of daily life—was not necessarily observed.

Among all participants, monthly forest visitors exhibited levels of experience of feeling happier, *ikigai*, and enjoyment of daily life that were approximately 14 percentage points higher and a sense of happiness that was approximately 9.8 percentage points higher than those who rarely visited forests.

### 3.3. Logistic Regression Analysis

Table 3 presents the results of the logistic regression analysis of Model 1, adjusted for sex in the case of total, age, smoking status, alcohol consumption, and habitual exercise. The frequency of forest walking was significantly associated with the experience of feeling happier, a sense of happiness, *ikigai*, and enjoyment of daily life among all participants and among women. Among men, the frequency of forest walking was significantly associated with experience of feeling happier, *ikigai*, and enjoyment of daily life.

Regarding having experience of feeling happier than usual, the adjusted odds ratios (aORs) (95% confidence intervals [CIs]) in the group that reported a frequency of forest walking of once a month or more were 1.73 (1.30–2.29) among all participants, 2.02 (1.27–3.19) among men, and 1.55 (1.09–2.23) among women when the group that reported rarely forest walking was used as the reference. While no statistically significant results were observed for the frequency of “once a year” among all participants or by gender, statistically significant odds ratios were observed for the frequency of “several times a year” among all participants and among women.

Regarding having a sense of happiness, the aORs (95% CIs) in the group that reported a frequency of forest walking of once a month or more were 1.56 (1.15–2.11) among all participants and 1.78 (1.18–2.67) among women when the group that reported rarely forest walking was used as the reference, and these results were statistically significant. The adjusted odds ratio for “once a year” was not statistically significant in either the overall participant group or the female group, but the adjusted odds ratio for “several times a year” was significant. In contrast, for men, none of the odds ratios for the frequency of forest walks were statistically significant.

Regarding having *ikigai*, the aORs (95% CIs) in the group that reported a frequency of forest walking of once a month or more were 1.68 (1.27–2.23) among all participants, 1.87 (1.16–3.01) among men, and 1.62 (1.14–2.30) among women when the group that reported rarely forest walking was used as the reference. With the exception of the adjusted odds ratio for women who went for a forest walk once a year, all other adjusted odds ratios were statistically significant.

Regarding enjoyment of daily life, the aORs (95% CIs) in the group that reported a frequency of forest walking of once a month or more were 2.38 (1.60–3.55) among all participants, 2.05 (1.09–3.83) among men, and 2.52 (1.50–4.24) among women when the group that reported rarely forest walking was used as the reference. For the overall population as well as for both men and women, this showed the highest odds ratio among the four outcomes. All adjusted odds ratios were statistically significant, even once a year, among all participants and among both men and women. The adjusted odds ratios were all statistically significant, even for the group that took a forest walk once a year, for all participants and for both men and women.

The results of these logistic regression analyses showed that the trends differed between men and women. In the overall participant group and, in most cases, among women, adjusted odds ratios for all four outcomes showed an upward trend with increasing frequency of forest walks. In contrast, among men, although the group that rarely visited forests consistently had the lowest proportion of positive responses for all four indicators, a relationship whereby the adjusted odds ratios, except for “experiences of feeling happier,” increased as the frequency of forest walking rose was not observed. This deviation from the relationship in which the proportion of positive responses increases with increasing frequency of forest walks was more pronounced in the logistic regression analysis than in the results presented in Table 2.

Table 4 reports the results of the logistic regression analysis for Model 2, which adds social factors such as employed status and years of education as independent variables, in addition to the lifestyle-related independent variables from Model 1. In the model, the results were similar to those of Model 1. Frequency of forest walking was again significantly associated with having experience of feeling happier, a sense of happiness, *ikigai*, and enjoyment of daily life in all, except having a sense of happiness among men.

## 4. Discussion

This study found that the frequency of forest walking was associated with happiness, *ikigai*, and enjoyment of daily life. The majority of the findings in this study indicated that groups engaging in forest walking more frequently tended to have higher levels of well-being and *ikigai*. This suggests that incorporating forest walking into daily life and visiting forests more often may contribute to improving overall well-being.

This study, which evaluated the effects of forest bathing through forest walking, represents a unique approach within epidemiological research. Meanwhile, over one hundred studies have evaluated the effects of green spaces on human health [39,40,41]. However, the association between green spaces and well-being is not exactly the same as the association between *shinrin-yoku* or forests and well-being, because green spaces do not necessarily require forests, whereas forest bathing requires a forest environment.

Furthermore, perceptions regarding the definition of green spaces and forests vary from country to country. In Japan, there are several definitions of green spaces and forests, some of which include forests within the category of green spaces [42]. That said, many Japanese people consider green spaces and forests to be distinct entities [43]. Green spaces are generally perceived as flat, open, and maintained areas, such as lawns and urban green spaces. In contrast, forests are perceived as natural environments typically located in mountainous regions.

From a global perspective, forests are generally regarded as part of green spaces. However, in many parts of the world, forests are not the primary natural environment and are not easily accessible. For this reason, studies conducted in other countries on the association between green spaces, natural environments, and health have presented definitions of green spaces and natural environments; however, forests do not constitute the majority of green spaces [12,39,40,41]. Therefore, while keeping in mind the differences among forests, green spaces, and natural environments, the present study compares and discusses its findings in relation to previous research on the association between well-being and green spaces and natural environments.

A sex difference was observed in this study. Previous research on the association between green spaces and mental well-being has also reported sex differences. Although the evaluation metrics differ from those used in this study, a review of urban green spaces and mental health has reported that women tend to benefit more [44]. Although this study cannot identify the cause of sex differences in the results, one possible explanation for the sex difference observed in this study is that, among men, only 87 participants reported engaging in forest walking once per year, which was smaller than that in the other groups; therefore, the results for this group may be unstable.

Although the definition of well-being differs from that used in this study, it has been reported that spending at least 120 min per week in natural environments leads to higher levels of well-being the following week [45]. A systematic review showed that green space has positive associations with mental well-being, and, in particular, there are associations between the amount of local-area green space and life satisfaction [46]. The World Health Organization also recommends that green spaces should be accessible within a 300 m linear distance of residences [47].

On the other hand, in this study, even forest walking only several times a year was significantly associated with well-being indicators, such as happiness, *ikigai*, and enjoyment of daily life, among all participants. Further, a frequency of forest walking of only once a year was significantly associated with *ikigai* and enjoyment of daily life among men and significantly associated with enjoyment of daily life among women. These results suggest even relatively infrequent forest walking (e.g., several times a year) may contribute to well-being, although more frequent engagement may yield greater benefits. Although this study could not identify the reasons for the differences in recommended frequency between green spaces in urban residential areas reported in previous studies and visits to forests in this study, one possible explanation is that visiting forested areas allows individuals to escape from daily routines and may offer a deeper experience of nature. Another possibility is that unknown confounding factors related to the frequency of forest walks may be associated with well-being.

It is feasible to recommend engaging in forest bathing through forest walking several times a year to improve health and well-being. At this frequency, it is possible for people to achieve this even if they avoid it during periods of severe weather, such as mid-summer or mid-winter. As approximately 67% of Japan’s land area is covered in forest, it is easy to access a forest within one hour of travel, with the exception of some areas (e.g., the center of Tokyo). The same is true for many of Japan’s megacities, including Nagoya. Although the percentage of forested areas in Nagoya itself is low, the percentage of forested areas in Aichi Prefecture, in which Nagoya is located, as well as in the neighboring prefectures of Gifu and Mie, is 44–81% [38].

Since forest bathing is a health-promoting activity that requires no special skills, it is easy to do. In Japan, guided forest bathing tours are extremely limited, and the vast majority of forest bathing is done by forest walks without a guide. This study demonstrates that even forest bathing through unguided forest walks can provide sufficient benefits. Based on the results of this study, it would be desirable for people to visit forests more often to improve their health. To summarize epidemiological studies on forest bathing through forest walks, beneficial effects have been reported on subjective and mental health–related outcomes, such as sleep, stress-coping ability, and well-being as assessed in this study. In contrast, no association with hypertension has been observed, suggesting that its effects on physical health may be limited [16,17,18,19]. Further research is needed to clarify its impact on physical health.

It has been reported that *ikigai* and well-being are associated with lower mortality and disease risk [32,33,34,48]. Therefore, it is conceivable that frequent forest bathing could enhance *ikigai* and well-being, which in turn might reduce the risk of death and disease. Although this study cannot test this hypothesis, it is hoped that it will be tested in future research.

This study has some limitations. First, no causal relationships could be identified, as this was a cross-sectional study. This cross-sectional study cannot determine whether frequent forest walking leads to increased happiness, *ikigai*, and enjoyment of daily life, or whether individuals who already experience higher levels of well-being are more likely to visit forests. Cohort studies are therefore warranted to evaluate the efficacy of forest walking for improving well-being in the future. Second, the research designs of epidemiological studies have limitations. For example, they cannot uncover the mechanisms through which forest walking as a habit contributes to daily well-being. In addition, detailed information such as what types of forests and forest walking methods are more effective cannot be obtained from epidemiological studies. Such considerations need to be verified in well-controlled intervention studies. Third, because the frequency of forest walks and all outcome measures are based on self-reports via a questionnaire, this may lead to response bias. Furthermore, questionnaire items used in this study regarding daily happiness, *ikigai*, and enjoyment of daily life were not standardized. Nevertheless, single-item measures such as “Do you have ‘*ikigai*’ in your life?” have been used and have also been shown to be associated with mortality [49,50]. Furthermore, having daily happiness, *ikigai*, and enjoyment of daily life are influenced by various social factors. While our questionnaire included hundreds of questions, the questions on social factors such as working status and years of education were extremely limited. For this reason, although we conducted multivariate analyses, the adjustment of social factors may have been insufficient.

Nevertheless, the strength of this study is that it examined whether forest bathing habitually has the potential to contribute to daily well-being—rather than only providing a temporary acute effect—based on epidemiological research with a large number of participants (over 3000).

As a future direction, causal relationships should be investigated in a cohort study, as the present cross-sectional design does not permit causal inference. The J-MICC Study is a cohort study that will continue tracking cancer incidence and mortality until 2025. Therefore, it is planned to verify whether the habit of forest bathing contributes to cancer prevention and reduced mortality. In addition, the participants in this study who voluntarily participated in the health survey are considered a group with a high level of health consciousness. In epidemiological studies, results may vary across populations; therefore, it is necessary to verify findings in other populations and to strengthen the evidence base through the accumulation of evidence.

## 5. Conclusions

This study in a general population of over 3000 Japanese individuals found that the frequency of forest bathing through forest walking was associated with happiness, *ikigai*, and enjoyment of daily life. Consequently, continuous forest walking may contribute to improvements in daily well-being, suggesting that even relatively infrequent forest walking (e.g., several times a year) may contribute to well-being. This study also demonstrated that forest bathing through forest walking may have beneficial effects even without guided or specialized programs. In this way, forest bathing requires no specialized skills and can be readily practiced by simply visiting forests; therefore, visiting forests several times a year may be recommended for health promotion.

As this study is cross-sectional, causal inference is limited, and several other limitations should also be acknowledged. Therefore, future studies should examine causal relationships using cohort designs, and efforts to verify the findings in other populations, along with the accumulation of evidence, are needed to enhance the validity and generalizability of the findings. This will contribute to the further establishment of scientific evidence regarding the health effects of forest bathing.

## Figures and Tables

**Table 1:** Characteristics of the participants by the frequency of forest walking.

Frequency of Forest Walking	Once a Month or More		Several Times a Year		Once a Year		Rarely		Total	*p* Value
	*n*	%	*n*	%	*n*	%	*n*	%	*n*	
Total	334	(9.6)	771	(22.2)	391	(11.3)	1976	(56.9)	3472	
Sex										
Men	132	(13.8)	223	(23.4)	87	(9.1)	512	(53.7)	954	<0.001 ^(b)^
Women	202	(8.0)	548	(21.8)	304	(12.1)	1464	(58.1)	2518	
Total										
Age (mean ± S.D.) (years)	62.4 ± 9.4		58.7 ± 10.3		57.9 ± 10.1		58.1 ± 9.8		58.7 ± 10.0	<0.001 ^(c)^
Alcohol consumption	150	(44.9)	352	(45.7)	142	(36.3)	727	(36.8)	1371	<0.001 ^(d)^
Smoking status	35	(10.5)	50	(6.5)	17	(4.3)	162	(8.2)	264	0.94 ^(d)^
Habitual exercise ^(a)^	268	(80.2)	524	(68.0)	236	(60.4)	1020	(51.6)	2048	<0.001 ^(d)^
Working	162	(48.5)	447	(58.0)	226	(57.8)	1202	(60.8)	2037	<0.001 ^(d)^
Years of education										0.006 ^(d)^
≤9	12	(3.6)	29	(3.8)	10	(2.6)	101	(5.1)	152	
10–12	122	(36.5)	264	(34.2)	128	(32.7)	748	(37.9)	1262	
13–15	99	(29.6)	228	(29.6)	124	(31.7)	580	(29.4)	1031	
>15	101	(30.2)	250	(32.4)	129	(33.0)	547	(27.7)	1027	
Men										
Age (mean ± S.D.) (years)	63.7 ± 9.2		60.3 ± 10.2		59.8 ± 10.0		59.5 ± 9.6		60.3 ± 9.8	0.054 ^(c)^
Alcohol consumption	84	(63.6)	160	(71.7)	57	(65.5)	302	(59.0)	603	0.01 ^(d)^
Smoking status	25	(18.9)	29	(13.0)	10	(11.5)	95	(18.6)	159	0.37 ^(d)^
Habitual exercise ^(a)^	105	(79.5)	150	(67.3)	51	(58.6)	271	(52.9)	577	<0.001 ^(d)^
Working	71	(53.8)	136	(61.0)	62	(71.3)	368	(71.9)	637	<0.001 ^(d)^
Years of education										0.40 ^(d)^
≤9	4	(3.0)	5	(2.2)	1	(1.1)	22	(4.3)	32	
10–12	45	(34.1)	66	(29.6)	25	(28.7)	162	(31.6)	298	
13–15	14	(10.6)	16	(7.2)	4	(4.6)	55	(10.7)	89	
>15	69	(52.3)	136	(61.0)	57	(65.5)	273	(53.3)	535	
Women										
Age (mean ± S.D.) (years)	61.5 ± 9.5		58.1 ± 10.2		57.3 ± 10.1		57.7 ± 9.9		58.0 ± 10.0	0.001 ^(c)^
Alcohol consumption	66	(32.7)	192	(35.0)	85	(28.0)	425	(29.0)	768	0.02 ^(d)^
Smoking status	10	(5.0)	21	(3.8)	7	(2.3)	67	(4.6)	105	0.66 ^(d)^
Habitual exercise ^(a)^	163	(80.7)	374	(68.2)	185	(60.9)	749	(51.2)	1471	<0.001 ^(d)^
Working	91	(45.0)	311	(56.8)	164	(53.9)	834	(57.0)	1400	0.03 ^(d)^
Years of education										0.10 ^(d)^
≤9	8	(4.0)	24	(4.4)	9	(3.0)	79	(5.4)	120	
10–12	77	(38.1)	198	(36.1)	103	(33.9)	586	(40.0)	964	
13–15	85	(42.1)	212	(38.7)	120	(39.5)	525	(35.9)	942	
>15	32	(15.8)	114	(20.8)	72	(23.7)	274	(18.7)	492	

^(a)^ Leisure time activity (once a week for 30 min or more); ^(b)^ chi-squared test; ^(c)^ linear regression model; ^(d)^ Mantel–Haenszel test for trend.

**Table 2:** Proportions of happiness, *ikigai*, and enjoyment of daily life based on the frequency of forest walking.

Frequency of Forest Walking	Once a Month or More		Several Times a Year		Once a Year		Rarely		Total	*p* Value ^(a)^
	*n*	%	*n*	%	*n*	%	*n*	%	*n*	
Total										
Experience of feeling happier	261	(78.1)	574	(74.4)	263	(67.3)	1270	(64.3)	2368	<0.001
A sense of happiness	275	(82.3)	623	(80.8)	303	(77.5)	1433	(72.5)	2634	<0.001
* ikigai*	261	(78.1)	581	(75.4)	282	(72.1)	1259	(63.7)	2383	<0.001
Enjoyment of daily life	304	(91.0)	664	(86.1)	334	(85.4)	1518	(76.8)	2820	<0.001
Men										
Experience of feeling happier	103	(78.0)	153	(68.6)	59	(67.8)	308	(60.2)	623	<0.001
A sense of happiness	104	(78.8)	173	(77.6)	70	(80.5)	365	(71.3)	712	0.03
* ikigai*	106	(80.3)	177	(79.4)	72	(82.8)	333	(65.0)	688	<0.001
Enjoyment of daily life	119	(90.2)	182	(81.6)	76	(87.4)	388	(75.8)	765	<0.001
Women										
Experience of feeling happier	158	(78.2)	421	(76.8)	204	(67.1)	962	(65.7)	1745	<0.001
A sense of happiness	171	(84.7)	450	(82.1)	233	(76.6)	1068	(73.0)	1922	<0.001
* ikigai*	155	(76.7)	404	(73.7)	210	(69.1)	926	(63.3)	1695	<0.001
Enjoyment of daily life	185	(91.6)	482	(88.0)	258	(84.9)	1130	(77.2)	2055	<0.001

^(a)^ Mantel–Haenszel test for trend.

**Table 3:** Adjusted odds ratios (aORs) of the frequency of forest walking for happiness, *ikigai*, and enjoyment of daily life.

	Experience of Feeling Happier		A Sense of Happiness		*Ikigai*		Enjoyment of Daily Life	
Frequency of Forest Walking	aOR ^(a)^	95% CI ^(b)^	aOR	95% CI	aOR	95% CI	aOR	95% CI
Total	*p* < 0.001		*p* < 0.001		*p* < 0.001		*p* < 0.001	
Once a month or more	1.73	(1.30–2.29)	1.56	(1.15–2.11)	1.68	(1.27–2.23)	2.38	(1.60–3.55)
Several times a year	1.55	(1.28–1.87)	1.47	(1.20–1.81)	1.59	(1.32–1.93)	1.65	(1.30–2.08)
Once a year	1.12	(0.89–1.41)	1.26	(0.97–1.63)	1.43	(1.12–1.82)	1.69	(1.24–2.29)
Rarely	1		1		1		1	
Men	*p* = 0.01		*p* = 0.31		*p* < 0.001		*p* = 0.03	
Once a month or more	2.02	(1.27–3.19)	1.26	(0.78–2.02)	1.87	(1.16–3.01)	2.05	(1.09–3.83)
Several times a year	1.36	(0.97–1.91)	1.25	(0.85–1.82)	1.91	(1.30–2.78)	1.18	(0.78–1.79)
Once a year	1.37	(0.84–2.24)	1.59	(0.90–2.81)	2.51	(1.39–4.53)	2.10	(1.06–4.15)
Rarely	1		1		1		1	
Women	*p* < 0.001		*p* < 0.001		*p* < 0.001		*p* < 0.001	
Once a month or more	1.55	(1.09–2.23)	1.78	(1.18–2.67)	1.62	(1.14–2.30)	2.52	(1.50–4.24)
Several times a year	1.64	(1.31–2.07)	1.58	(1.23–2.03)	1.50	(1.20–1.88)	1.93	(1.44–2.58)
Once a year	1.06	(0.81–1.38)	1.20	(0.89–1.60)	1.26	(0.96–1.64)	1.61	(1.15–2.27)
Rarely	1		1		1		1	

^(a)^ aOR: Adjusted odds ratio; ^(b)^ CI: Confidence interval; adjusted by sex (in the case of total), age (≤49, 50s, 60s, ≥70 (years)), smoking status (current smoker/other responses), alcohol consumption (once a week or more/other responses), and habitual exercise (leisure time activity: once a week for at least 30 min/other responses).

**Table 4:** Adjusted odds ratios (aORs) of the frequency of forest walking for having experience of daily happiness, *ikigai*, and enjoyment of daily life.

	Experience of Feeling Happier		A Sense of Happiness		*Ikigai*		Enjoyment of Daily Life	
Frequency of Forest Walking	aOR ^(a)^	95% CI ^(b)^	aOR	95% CI	aOR	95% CI	aOR	95% CI
Total	*p* < 0.001		*p* < 0.001		*p* < 0.001		*p* < 0.001	
Once a month or more	1.72	(1.29–2.28)	1.54	(1.14–2.09)	1.65	(1.25–2.20)	2.38	(1.60–3.55)
Several times a year	1.55	(1.28–1.87)	1.47	(1.19–1.81)	1.58	(1.30–1.91)	1.66	(1.31–2.10)
Once a year	1.12	(0.88–1.41)	1.26	(0.97–1.63)	1.41	(1.11–1.81)	1.71	(1.26–2.32)
Rarely	1		1		1		1	
Men	*p* = 0.01		*p* = 0.28		*p* < 0.001		*p* = 0.03	
Once a month or more	2.01	(1.27–3.19)	1.25	(0.78–2.01)	1.90	(1.18–3.08)	2.06	(1.10–3.86)
Several times a year	1.40	(0.99–1.97)	1.28	(0.88–1.88)	2.03	(1.38–2.99)	1.23	(0.81–1.87)
Once a year	1.42	(0.87–2.33)	1.60	(0.90–2.84)	2.55	(1.41–4.64)	2.14	(1.08–4.25)
Rarely	1		1		1		1	
Women	*p* < 0.001		*p* < 0.001		*p* = 0.001		*p* < 0.001	
Once a month or more	1.55	(1.08–2.22)	1.77	(1.18–2.65)	1.57	(1.10–2.23)	2.53	(1.50–4.25)
Several times a year	1.65	(1.31–2.07)	1.58	(1.23–2.03)	1.47	(1.17–1.83)	1.94	(1.45–2.60)
Once a year	1.05	(0.80–1.37)	1.19	(0.89–1.60)	1.23	(0.94–1.62)	1.64	(1.16–2.31)
Rarely	1		1		1		1	

^(a)^ aOR: Adjusted odds ratio; ^(b)^ CI: Confidence interval; adjusted by sex (in the case of total), age (≤49, 50s, 60s, ≥70 (years)), smoking status (current smokers/other responses), alcohol consumption (once a week or more/other responses), habitual exercise (leisure time activity: once a week for at least 30 min or more/other responses), working status, and years of education.

## Data Availability

The original contributions presented in this study are included in the article. Further inquiries can be directed to the corresponding author.

## Abbreviations

QOL: Quality of Life; J-MICC Study: Japan Multi-Institutional Collaborative Cohort Study; aOR: Adjusted Odds Ratio; CI: Confidence Interval.
